# Tumor infiltrating neutrophils and gland formation predict overall survival and molecular subgroups in pancreatic ductal adenocarcinoma

**DOI:** 10.1002/cam4.3695

**Published:** 2020-12-28

**Authors:** Julia R. Naso, James T. Topham, Joanna M. Karasinska, Michael K.C. Lee, Steve E. Kalloger, Hui‐li Wong, Jessica Nelson, Richard A. Moore, Andrew J. Mungall, Steven J.M. Jones, Janessa Laskin, Marco A. Marra, Daniel J. Renouf, David F. Schaeffer

**Affiliations:** ^1^ Division of Anatomic Pathology Vancouver General Hospital Vancouver BC Canada; ^2^ Department of Pathology and Laboratory Medicine University of British Columbia Vancouver BC Canada; ^3^ Pancreas Centre BC Vancouver BC Canada; ^4^ Division of Medical Oncology BC Cancer Vancouver BC Canada; ^5^ Canada's Michael Smith Genome Sciences Centre Vancouver BC Canada; ^6^ Department of Medical Genetics University of British Columbia Vancouver BC Canada

**Keywords:** molecular, pancreatic neoplasms, pathology, pathology, prognosis

## Abstract

**Background:**

RNA‐sequencing‐based classifiers can stratify pancreatic ductal adenocarcinoma (PDAC) into prognostically significant subgroups but are not practical for use in clinical workflows. Here, we assess whether histomorphological features may be used as surrogate markers for predicting molecular subgroup and overall survival in PDAC.

**Methods:**

Ninety‐six tissue samples from 50 patients with non‐resectable PDAC were scored for gland formation, stromal maturity, mucin, necrosis, and neutrophil infiltration. Prognostic PDAC gene expression classifiers were run on all tumors using whole transcriptome sequencing data from the POG trial (NCT02155621). Findings were validated using digital TCGA slides (n = 50). Survival analysis used multivariate Cox proportional‐hazards tests and log‐rank tests.

**Results:**

The combination of low gland formation and low neutrophil infiltration was significantly associated with the poor prognosis PDAC molecular subgroup (basal‐like or squamous) and was an independent predictor of shorter overall survival, in both frozen section (n = 47) and formalin‐fixed paraffin‐embedded (n = 49) tissue samples from POG patients, and in the TCGA samples. This finding held true in the subgroup analysis of primary (n = 17) and metastatic samples (n = 79). The combination of high gland formation and high neutrophils had low sensitivity but high specificity for favorable prognosis subgroups.

**Conclusions:**

The assessment of gland formation and neutrophil infiltration on routine histological sections can aid in prognostication and allow inferences to be made about molecular subtype, which may help guide patient management decisions and contribute to our understanding of heterogeneity in treatment response.

## Graphical abstract

The combination of low gland formation and low neutrophils is significantly associated with poor prognosis molecular subgroups of pancreatic ductal adenocarcinoma, and is an independent predictor of shorter overall survival. These features can be readily assessed in archival tissue slides, allowing inferences regarding molecular subtype and prognosis to be made as part of routine patient care.

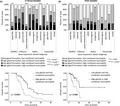

## 1 INTRODUCTION

Pancreatic ductal adenocarcinoma (PDAC) is the third leading cause of cancer death in the United States, with a 5 year survival of only 9%.^1^ Variability in the outcome of individual patients has been linked to differences in the activity of particular gene expression programs. Gene expression‐based molecular classifiers, including those proposed by Moffitt et al.,^2^ Collisson et al.,^3^ Bailey et al.,^4^ and Karasinska et al.,^5^ stratify PDAC patients into prognostically significant subgroups. The poor prognosis subgroups in these classifiers (i.e., the basal‐like, quasi‐mesenchymal, squamous, and glycolytic subgroups, respectively) show significant overlap, sharing signatures of endodermal identity loss and epigenetic dysregulation.^4^, ^6^, ^7^ Going forward, these molecular classifiers provide frameworks through which to study heterogeneity in the factors that drive disease progression.

As molecular subtypes had prognostic significance independent of clinical prognostic factors such as grade and stage,^2^, ^3^, ^4^, ^5^ the application of molecular classifiers to individual tumors has the potential to further refine prognostication and inform patient management. Early evidence suggests that PDAC classical subtype is associated with increased sensitivity to FOLFIRINOX treatment in the metastatic setting,^8^, ^9^ such that molecular stratification of tumors may also play a future role in treatment selection. However, gene expression analysis is not currently a routine part of clinical workflows. The incorporation of information from molecular classifiers into clinical practice, therefore, relies on the development of surrogate markers.

*GATA6* expression detected through situ hybridization or immunohistochemistry has shown utility in predicting classical versus basal‐like molecular subtype,^9^ but cannot be performed retrospectively without access to tissue, adds time and expense, consumes valuable tissue, and requires reagents not routinely available in most clinical laboratories. A practical alternative for a surrogate marker of molecular subtype may be histological features scored during routine assessment of tumor tissue or retrospectively evaluated in archival H&E slides. While tumor stage and grade were correlated with particular subtypes, in our opinion these factors do not stratify well enough to be used as surrogate markers for molecular subtype. For instance, stage I/II tumors were 62% classical subtype, whereas than Stage IV tumors were 46% classical subtype.^10^ Grade 3 tumors were enriched in a subset of basal‐like tumors (~40% in “pure‐basal” vs. ~10–20% in other subgroups) and grade 1 tumors were enriched in a subset of classical tumors (~50% in “pure‐classical” vs. ~20–40% in other subgroups).^11^ Other subgroups showed intermediate proportions of each grade, and grade 2 tumors had a similar incidence across subgroups.^11^

Other histologic factors such as stromal volume,^12^ stromal maturity,^12^, ^13^, ^14^ gland formation,^15^ tumor budding,^16^ mucin content,^2^ and immune cell populations^17^, ^18^, ^19^ have been associated with overall survival, but require external validation or assessment for correlation with molecular subgroups. Most studies seeking to identify histologic correlates have focused on either architectural features or immune cell infiltration, without assessment of combinations of these features. Moreover, studies of immune cell infiltrates have largely relied on immunohistochemistry rather than cell morphology on H&E sections. Our understanding of whether prognostic features identified in primary tumors are relevant in metastatic tumors, and vice versa, is also limited, such that markers identified using one type of sample may not be valid on another.

Histological features may not only be a practical surrogate for gene expression, but also they may provide insight into to the cellular processes characteristic of subgroups. We note that molecular subtype nomenclature reflects dysregulation of particular gene expression programs, and not necessarily the histologic features of these tumors. For instance, even though adenosquamous tumors were most likely to be in the “squamous” molecular subgroup, the majority of tumors in the “squamous” molecular subgroup were PDAC “not otherwise specified”.^4^ Histological assessment can also inform on the spatial relationship of cell populations in a manner that sequencing cannot, a factor that may be particularly relevant to the characterization of immune cell infiltrates. Scoring of immune infiltrates in select compartments may reveal associations with survival that are not evident from sequencing a whole tissue core.

We sought to identify novel histological correlates of molecular subtypes and overall survival that could be scored on routine sections of primary and metastatic tumors, and thereby directly applied to clinical practice. Moreover, we sought to address the questions of whether histologic features may have greater prognostic significance in combination than individually, whether histomorphological scoring of immune infiltrates may have prognostic value, whether tissue from primary and metastatic sites can be interpreted in the same manner to predict molecular subtype prediction, and whether scoring in particular tumor compartments (i.e., tumor stroma vs. tumor gland lumens) may improve prognostic significance. We here report the novel prognostic significance and molecular subgroup correlates of histologically identifiable neutrophil infiltrates, and show that overall survival is stratified by combined neutrophil and gland formation scores.

## 2 MATERIALS AND METHODS

All “in‐house” cases were from patients with non‐resectable PDAC who were enrolled in the Personalized Oncogenomics (POG, NCT02155621) and/or PanGen (NCT02869802) trials at British Columbia Cancer. Each patient provided written informed consent prior to sequencing. Whole genome and transcriptome sequencing was performed as described previously,^20^ and is accessible through the European Genome‐468 phenome Archive (EGA; http://www.ebi.ac.uk/ega/) under study accession number #EGAS00001001159. This study was approved by the University of British Columbia Research Ethics Committee (REB# H12‐00137, H14‐00681, H16‐00291) and conducted in accordance with international ethical guidelines.

Archival frozen section and/or formalin‐fixed paraffin‐embedded (FFPE) tissue was retrospectively identified from 50 patients with “in‐house” sequenced tumors. All frozen section slides contained tissue from the samples used for sequencing, which were obtained from 40 liver metastases, 3 supraclavicular lymph nodes, 2 peritoneal nodules, and 2 primary tumors. FFPE sections were clinical samples obtained from 27 liver metastases, 15 primary tumors, 3 left supraclavicular masses, 2 gastrointestinal metastases, 1 intraperitoneal lymph node, and 1 tongue metastasis. All specimens were small biopsies except for 7 pancreaticoduodenectomies, 2 distal pancreatectomies, and 1 bowel resection. Digital slide images and clinical data for an additional 50 primary PDAC cases from The Cancer Genome Atlas (TCGA)^21^ were accessed through the National Cancer Institute Genomic Data Commons Data Portal (https://portal.gdc.cancer.gov/). All 25 interpretable (i.e., adequate image quality and tumor content >5%) basal subtype TCGA cases and an equal number of randomly selected classical subtype TCGA cases were scored. Moffitt subtypes were determined using the RNA‐sequencing‐based PurIST algorithm.^22^ Collisson, Bailey, and Karasinska subtypes were determined as previously described.^5^ An empirical Bayesian approach^23^ was used to correct RNA‐seq gene expression data for cohort‐specific batches (POG and TCGA). Alleviation of inter‐sample batch effects after correction was confirmed using principal component analysis of the top 10% most variable genes.

**FIGURE 1:**
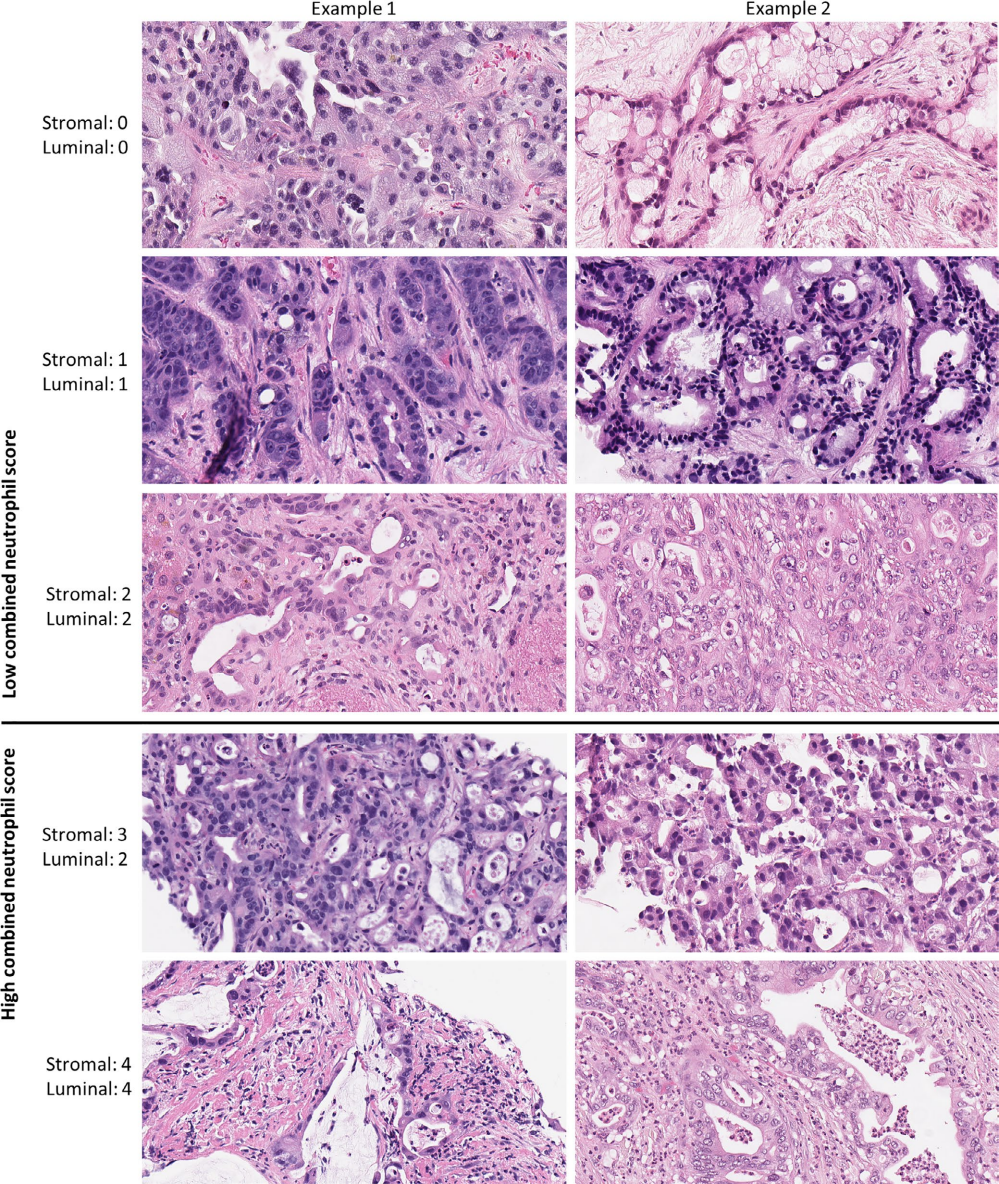
Representative images of FFPE in‐house samples with different neutrophil infiltration scores. Cases scoring ≤2 out of 4 for both stromal and luminal neutrophils were considered to have “low combined neutrophils.” H&E, X200.

All hematoxylin and eosin stained (H&E) sections from each tissue sample were reviewed for diagnosis and scored blinded to the subgroup and survival data. Gland formation was scored according to previously published criteria.^15^ Neutrophil density was scored on a five level scale ranging from 0 (none) to 4 (heavy infiltrate), based on which reference image (Figure 1, Figure S1) best represented the highest neutrophil density in one 200x field of the specimen. Neutrophils in an open space surrounded by tumor were considered luminal neutrophils, even if the tumor architecture did not meet criteria for gland formation. Stromal neutrophils were scored in the stroma immediately surrounding tumor cells. Stromal maturity was scored as either entirely immature (fibrotic stroma with myxoid changes) or partially mature (having even focal areas of keloid‐like or fine mature collagen), similar to published criteria.^24^ Necrosis and mucin production were scored as either present or absent.

Fisher's exact test was used for comparisons of categorical variables, whereas two‐tailed Wilcoxon–Mann–Whitney tests and Spearman correlation coefficients were used for comparisons of continuous variables. Cox proportional‐hazards tests and Kaplan–Meier curves with log‐rank test *p*‐values were generated using the “survival” (version 3.1–8)^25^ and “survminer” (version 0.4.6)^26^ packages in RStudio (version 1.2.1335). Multivariate analysis for in‐house samples included patient age and sex, tumor grade, extent of disease at diagnosis (metastatic versus locally advanced), whether the primary tumor was resected and whether the patient received immunotherapy. Multivariate analysis for TCGA samples included patient age at diagnosis and sex. *p*‐values less than 0.05 were considered significant.

## 3 RESULTS

**FIGURE 2:**
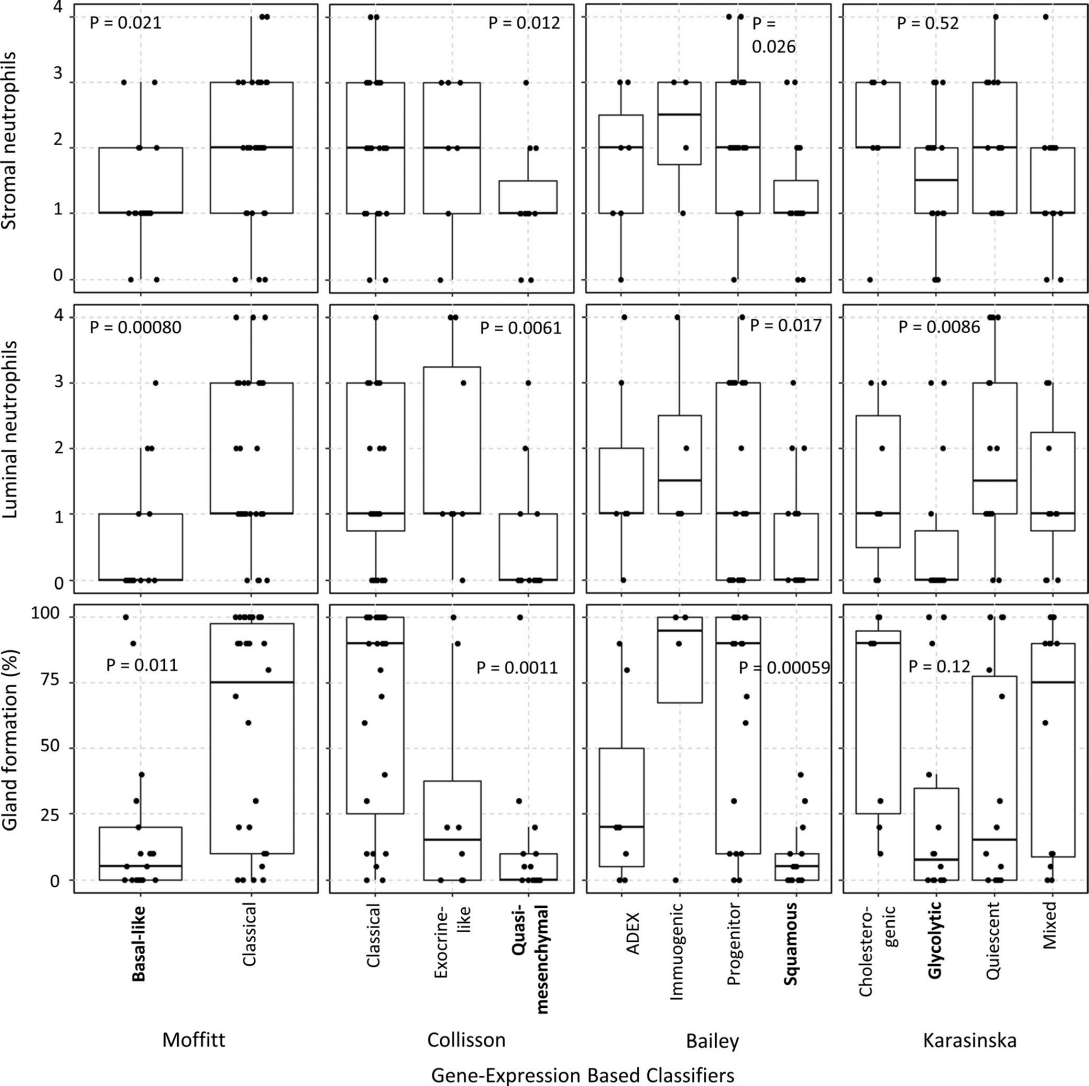
When using the frozen section in‐house samples (n = 47), neutrophil infiltration and gland formation were significantly associated with basal‐like, quasi‐mesenchymal, and squamous subtypes. *P*‐values were calculated using the Wilcoxon–Mann–Whitney test to compare the poor prognosis subgroup (shown in bold) to the other subgroups. Boxes extend from the first to third quartile, with a line at the median. Points indicate scores for individual samples.

We identified 50 non‐resectable PDAC patient samples with sequencing and histology available (Table S1). We first assessed the frozen tissue sections (n = 47) prepared from the samples used for sequencing (referred to as the in‐house frozen section samples), as these samples may best reflect the biology captured in sequencing data. Of the scored histologic features, low neutrophil infiltration (both stromal and luminal) and low gland formation were significantly associated with poor prognosis Moffitt, Collisson, and Bailey subtypes (basal‐like, quasi‐mesenchymal, and squamous, respectively, Figure 2). Stromal maturity, necrosis, and mucin were not significantly associated with any molecular subtypes (Table S2).

**FIGURE 3:**
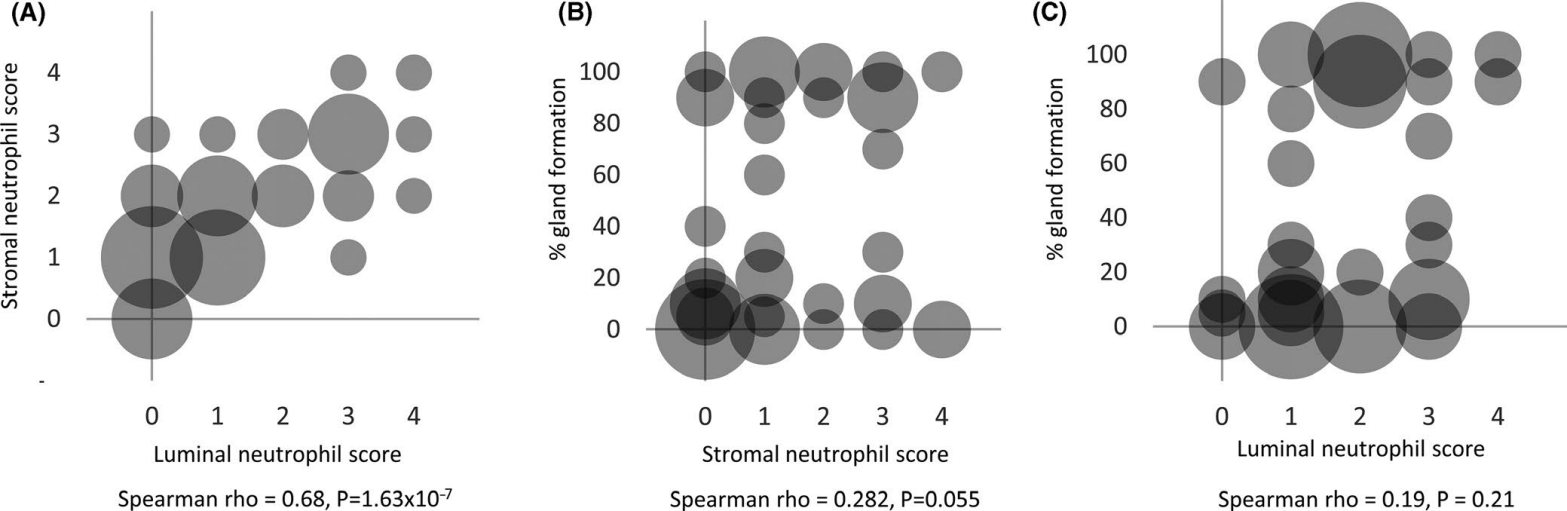
Correlation between percent gland formation, stromal neutrophil score, and luminal neutrophil score in the same sample, using frozen section in‐house validation samples. (A) Stromal and luminal neutrophil scores were well correlated, whereas (B,C) gland formation was poorly correlated with neutrophil scores. Bubble size is proportional to the number of case with a given score combination, ranging up to 8 for (A), up to 5 for (B), and up to 6 for (C). Spearman correlation (rho) and *P*‐values are shown.

**FIGURE 4:**
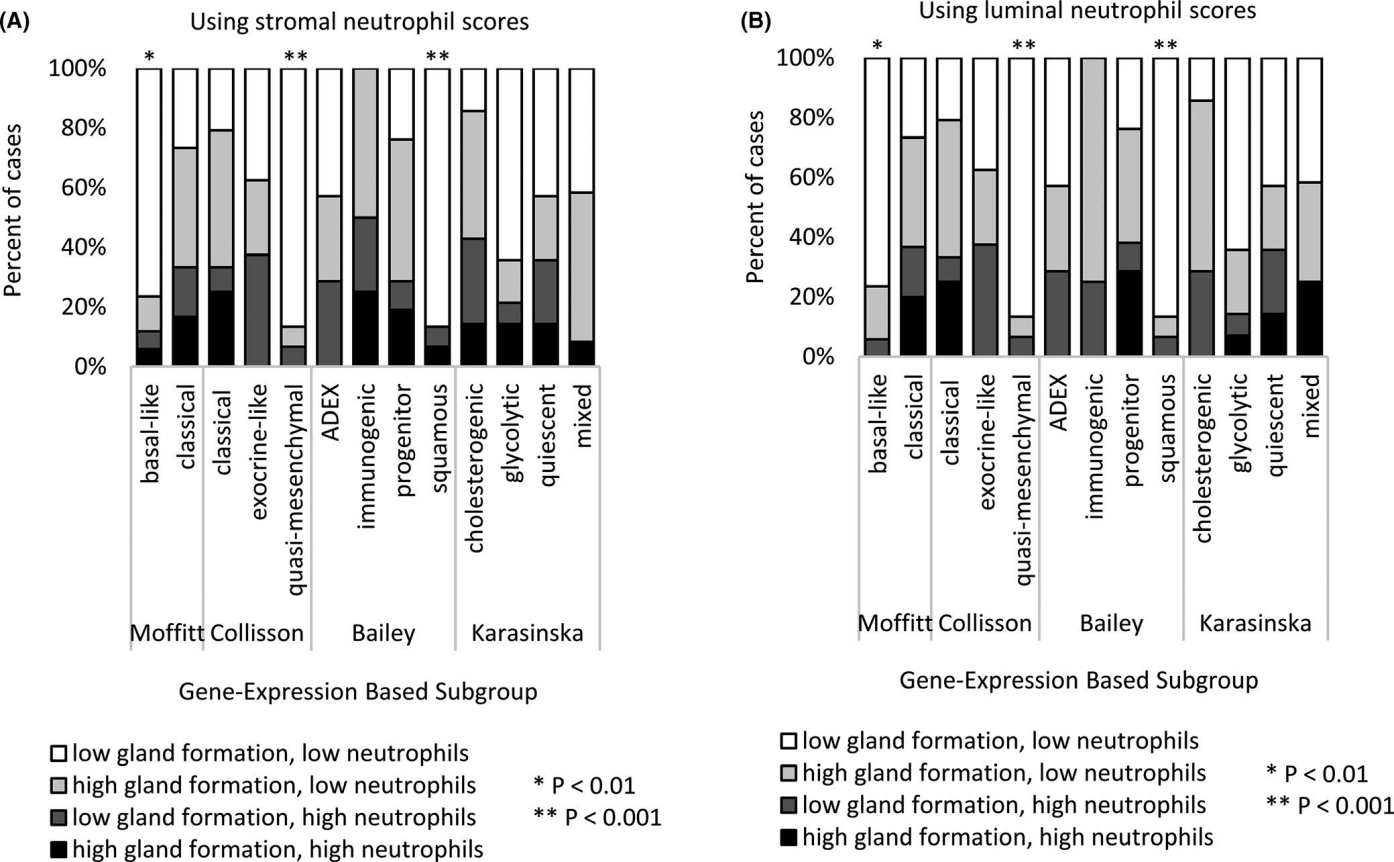
The proportion of samples with low gland formation and low neutrophils (either [A] stromal or [B] luminal) in each subgroup of the gene expression‐based classifiers, scored using the frozen section in‐house samples (n = 47). Cases with both low gland formation and low neutrophils were significantly enriched in basal‐like, quasi‐mesenchymal, and squamous subtypes. Fisher's exact test *P*‐values are shown. Low gland formation was defined as ≤30% and low neutrophils as a score of ≤2 out of 4.

Luminal and stromal neutrophil scores were moderately correlated (Spearman rho = 0.68, *p* = 1.63 × 10^−7^, Figure 3B). In contrast, neutrophil infiltration and gland formation were not significantly associated (Spearman rho ≥0.19, *p* ≥ 0.055; Figure 3C. Defining low gland formation as ≤30% and low neutrophil infiltration as ≤2 out of 4 (thresholds that optimally divided the data), the presence of both low gland formation and low neutrophils (stromal or luminal) tended to be more strongly associated with basal‐like, quasi‐mesenchymal, and squamous subtypes than each of these features alone (Figure 4, Table S2).

**FIGURE 5:**
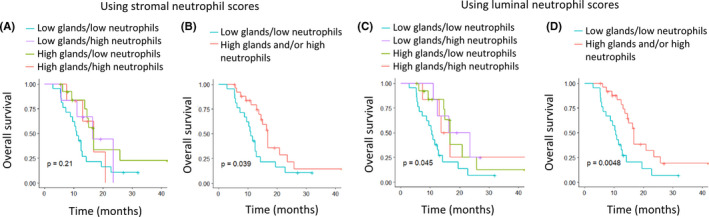
Kaplan–Meier curves for OS stratified by tumor gland formation and neutrophil infiltration, scored in frozen section in‐house samples. Low gland formation was defined as ≤30% and low neutrophils as a score of ≤2 out of 4. *P*‐values were calculated using log‐rank tests.

We then assessed whether scoring neutrophil infiltration and gland formation was sufficient to predict overall survival (OS). Patients whose tumors had low neutrophils (stromal or luminal) and low gland formation had significantly shorter OS compared to patients whose tumors had higher levels of either of these features (using stromal neutrophils: 11.0 vs. 16.8 months median OS; using luminal neutrophils: 10.7 vs. 16.8 months median OS; Figure 5; Table S3). There were no significant differences between groups in the type of first‐line chemotherapy used (i.e., fluorouracil/oxaliplatin vs. gemcitabine/paclitaxel based, *p* = 1), or in whether patients received immunotherapy (*p* > 0.10). In multivariate analysis, the combination of low luminal neutrophils and low gland formation was significantly associated with shorter OS, independent of age, sex, grade, extent of disease at diagnosis and whether the patient underwent resection or immunotherapy (hazard ratio 4.38, *p* = 0.016 Table S3).

We then sought to validate our findings using an independently scored separate set of FFPE tissue samples from 37 patients (referred to as the FFPE in‐house validation samples), of which 32 were taken on a different day from the same patient as one of the frozen section biopsies already assessed (median 12 weeks apart), and 3 were from additional patients without interpretable frozen sections. Up to three different FFPE samples from each patient were included, for a total of 49 FFPE validation samples. Fifteen of the 49 FFPE sections were from a different body site than a frozen section sample.

In the validation samples, low neutrophil infiltration and low gland formation tended to be associated with basal‐like, squamous, and quasi‐mesenchymal subtypes (*p* < 0.05 in eight out of nine comparisons, Figure S2). The combination of both low neutrophil infiltration (stromal or luminal) and low gland formation again tended to show a stronger association with the gene‐expression‐based classifiers (Figure S3, Table S2) and OS (using stromal neutrophils: 12.5 vs. 22.8 months median OS; using luminal neutrophils: 11.0 vs. 16.8 months median OS; Table S3, Figure S4) than these features analyzed separately. In multivariate analysis, the combination of low stromal or luminal neutrophils with low gland formation had independent prognostic significance (stromal: hazard ratio 3.51, *p* = 0.0041; luminal: hazard ratio: 3.07 *p* = 0.040; Table S3).

**FIGURE 6:**
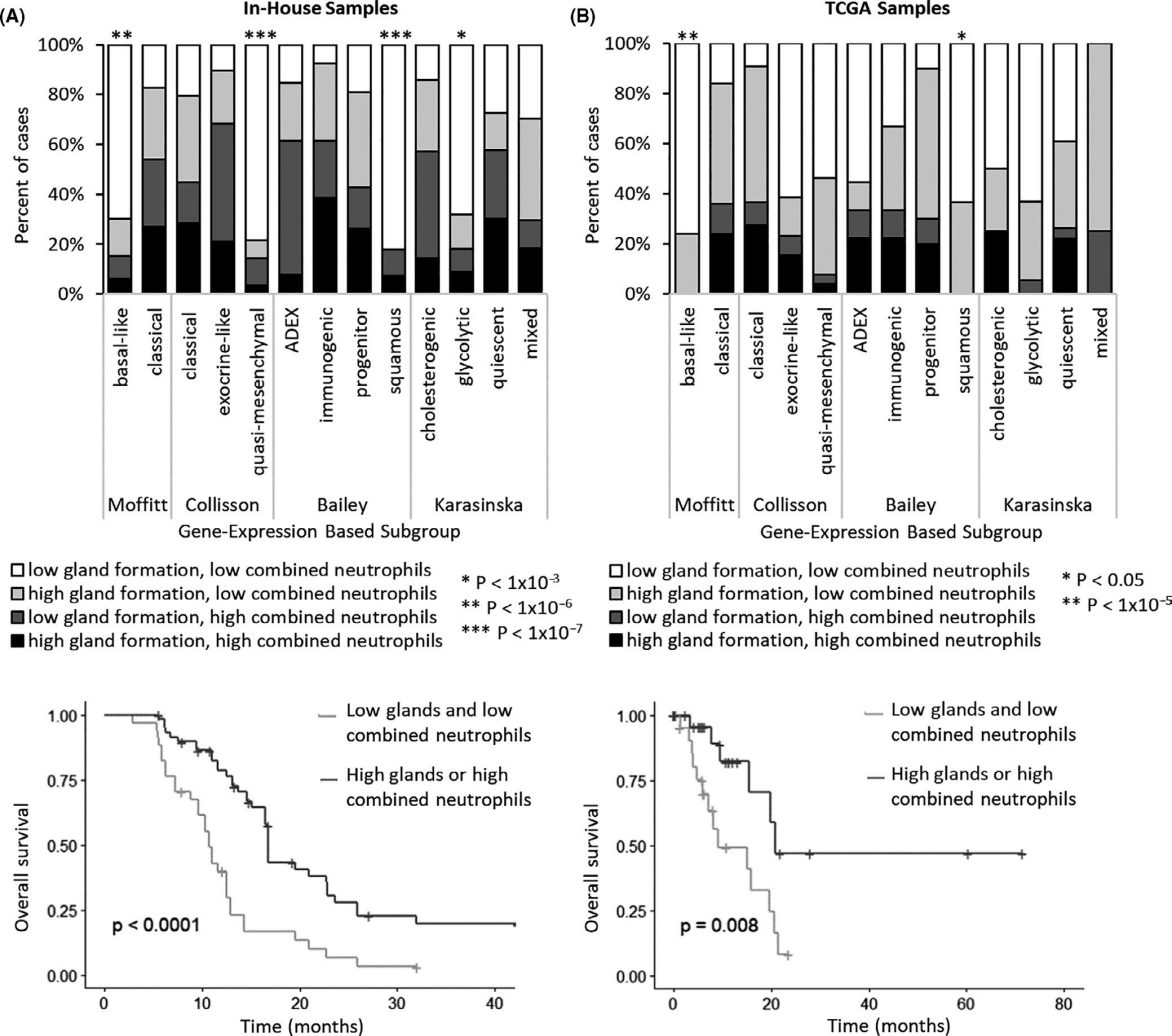
Subgroup and survival associations of low gland formation and low combined neutrophils in (A) pooled frozen and FFPE in‐house samples (n = 96) and (B) TCGA samples (n = 50). The top panels show the proportion of samples with low gland formation and low combined neutrophils in each subgroup of the gene expression‐based classifiers. Fisher's exact test *P*‐values are shown. The bottom panels show Kaplan–Meier curves for OS stratified by tumor gland formation and combined neutrophils. *P*‐values were calculated using log‐rank tests. Low gland formation was defined as ≤30%. Cases scoring ≤2 out of 4 for both stromal and luminal neutrophils were considered to have “low combined neutrophils.”

As luminal and stromal neutrophils remained well correlated (Spearman rho = 0.72, Figure S5), we simplified neutrophil scoring into one score: only cases with low stromal and luminal neutrophils were considered “low combined neutrophils,” and the remainder were considered “high combined neutrophils” (Figure 1). Reassessing the combined frozen and FFPE data, the association of low glands and low combined neutrophils with poor prognosis subgroups (all *p* < 1 × 10^−3^) and poor OS (10.7 vs. 16.8 months median OS, multivariate hazard ratio 2.48, Figure 6A, Table S4) remained significant, even in subgroup analyses of only samples of primary (n = 17) or metastatic (n = 79) disease (Figure 6A, Table S4). There remained no significant differences between groups in the type of first‐line chemotherapy used (i.e., fluorouracil/oxaliplatin vs. gemcitabine/paclitaxel based, *p* = 1), or in whether patients received immunotherapy (*p* > 0.22). The sensitivity and specificity of finding both low gland formation and low combined neutrophils was greatest for the squamous subgroup (82% sensitivity and 84% specificity; Table S5). The combination of high gland formation and high combined neutrophils, although not significantly associated with survival (multivariate Cox regression model *p* = 0.48, log‐rank test *p* = 0.14) and not sensitive for favorable prognosis subgroups (sensitivity only 23–27%), was highly specific for the favorable prognosis subgroups (specificity 91–96%, Table S6).

To validate these associations in an orthogonal patient cohort, we scored neutrophils and gland formation in TCGA primary resection samples (n = 50). Cases with both low gland formation and low combined neutrophils again had significantly shorter OS (9.1 vs. 20.8 months median OS; multivariate hazard ratio 3.49, Figure 6B, Table S4) and were significantly associated with the basal‐like (*p* = 4.38 × 10^−5^; sensitivity 76%, specificity 84%) and squamous (*p* = 0.045; sensitivity 64%, specificity 68%) subtypes (Figure 6B, Table S5). The combination of high gland formation and high combined neutrophils continued to have high specificity (96–100%) but low sensitivity (19–24%) for favorable prognosis subgroups (Table S6).

We then assessed gene expression signatures potentially associated with neutrophil infiltration in the in‐house and TCGA transcriptome sequencing data. Neutrophil‐associated gene expression was significantly higher in samples with a luminal or stromal neutrophil score of 4 (luminal: *p* = 0.014, stromal: *p* = 0.038; Figure S7A) but was not significantly associated with Moffitt molecular subtype (*p* = 0.47; Figure S7B). Of three genes recently implicated in neutrophil recruitment in PDAC, *TP63*, *IL1A*, and *CXCL1*,^27^ only increased *CXCL1* was significantly associated with luminal neutrophil score (*p* = 0.0091; Figure S7C). *TP63* and *IL1A* showed significantly higher expression in poor prognosis molecular subtype tumors (*TP63* *p* = 7.7 × 10^−6^; *IL1A* *p* = 0.0028), as expected from literature,^4^, ^27^ but *CXCL1* expression was not associated with molecular subtype (*CXCL1* *p* = 0.95; Figure S7D).

## 4 DISCUSSION

Gene‐expression profiling can stratify pancreatic adenocarcinoma into prognostic subgroups, but is not practical for incorporation into clinical workflow. Through a histomorphological assessment of immune cell infiltrates (in contrast to prior studies relying on immunohistochemistry) and assessment of novel combinations of histologic features, we propose a system for inferring molecular subtype using only routinely prepared tissue slides (i.e., no need for fresh frozen tissue, FFPE tissue blocks or immunohistochemistry). We show for the first time that the combination of low neutrophil infiltration and low gland formation is significantly associated with molecular subtypes and is an independent predictor of shorter overall survival. The opposite combination, high neutrophil infiltration and high gland formation, had high specificity for favorable prognosis subgroups, such that the presence of these features may be used to rule out poor prognosis subtypes. Scoring of these features may inform patient management decisions and may serve as a surrogate for molecular subtypes in clinical trials and translational research, contributing to our understanding of heterogeneity in treatment response.

The association of neutrophil infiltration and gland formation with molecular subtype proved robust across in‐house and external (TCGA) cases, frozen tissue and FFPE, primary and metastatic sites, and digital and glass slides, such that our findings are generalizable to a wide variety of sample types. Interestingly, histologic features scored in the FFPE samples were well correlated with molecular subgroups determined using sequencing data from the frozen section samples, despite differences in the timing and anatomical location of sampling between these sample groups. These data are consistent with the notion that subtype is most often retained over time and between tumor deposits in a patient. Instances of subtype plasticity have been noted in mouse models of PDAC,^8^, ^28^, ^29^ and a human study found two out of 11 matched primary and metastasis pairs to have discordant Moffitt subtypes.^30^ Another study found that two of six patients with PDAC samples at different time points had a change in molecular subtype associated with disease progression.^10^

The basal‐like, quasi‐mesenchymal, and squamous groups (i.e., the “poor prognosis” subgroups of different classifiers) are known to identify overlapping groups of patients.^4^, ^6^ These “poor prognosis” subgroups all show enrichment for mutations in chromatin modifiers, which are thought to mediate a loss of endodermal identity.^4^, ^6^, ^7^ Reduced gland formation in the poor prognosis subgroups may be a reflection of this underlying dedifferentiation. The more favorable prognosis subgroups also show overlap, with the Bailey immunogenic and progenitor groups potentially representing a subdivision of the Collisson and Moffitt classical groups.^6^ Similarly, Puleo et al. proposed subdivision of the classical group into “immune classical” and “pure classical” based on an immune infiltrate signature characteristic of the former.^11^ The prominent immune cell signature in some but not all classical tumors is in keeping with our finding that high neutrophil infiltration was specific to a subset of classical subgroup tumors. The classifier proposed by Karasinska et al. was the only classifier examined that was not significantly associated with neutrophil and gland formation levels. Unlike the other classifiers, the Karasinska classifier is based on the expression of genes associated with metabolic pathways, whose functions may have less impact on gland formation and immune cell recruitment than the genes used for other classifiers.

In line with the notion that prognosis is dependent on both tumor cell behavior and antitumor immune response, our assessment of combined gland formation and neutrophil infiltration integrates both of these components: gland formation may be an indicator of tumor differentiation and neutrophils may be a reflection of the immune microenvironment. Gland formation has previously been proposed to correlate with overall survival, though the optimal threshold in our data was 30% rather than the previously suggested 40%.^15^ The present study exemplifies how a combination of histologic features may be more strongly associated with molecular subtype and prognosis than any one histologic feature alone.

Mechanisms have been proposed through which neutrophils may have pro‐ or anti‐tumorigenic effects: Pro‐tumorigenic effects may be through inhibition of cytotoxic T‐lymphocyte activity, whereas anti‐tumorigenic effects may be through antibody‐dependent cytotoxicity and pro‐inflammatory cytokine production.^31^, ^32^, ^33^ Consistent with the association that we find between high neutrophil infiltration and favorable prognosis subgroups, pancreatic adenocarcinomas with high levels of CD66b positive cells (which includes both mature neutrophils and myeloid derived suppressor cells) and high CD20 positive B‐cells were shown in one study to have longer overall survival.^19^ However, other pancreatic adenocarcinoma studies have implicated neutrophils in shorter survival: high levels of CD66b positive tumor infiltrating cells were associated with shorter overall survival,^18^ poor prognosis “squamous” subtype tumors were shown to recruit neutrophils via a p63‐dependent mechanism,^27^ and depletion of CXCR2 or Ly6G positive cells (which includes neutrophils and myeloid derived suppressor cells) suppressed metastasis.^29^

Conflicting results between studies may be attributable in part to differences in how immune cell populations are defined (e.g., which genes or immunohistochemical markers are used) and in part to the local microenvironment in which immune cells are assessed. For instance, CXCR2 expression, which promotes myeloid cell recruitment, was associated with poor outcome when tumor edges were examined, but not when central tumor areas were examined.^29^ Such differences in the spatial location and types of immune cells detected may explain why in our study histologically scored neutrophils were significantly associated with subtype, but neutrophil‐associated gene expression was not: the latter reflects patterns across all sequenced tissue, whereas histologic scoring was focused on tumor nests and the immediately surrounding stroma. Moreover, neutrophil‐associated gene sets can be expected to have imperfect sensitivity and specificity for morphologically identifiable neutrophils. We also note that neutrophil abundance may be independent from pro‐tumorigenic neutrophil activity depending on the local microenvironment: it is possible that the smaller numbers of neutrophils in poor prognosis groups may be more able to contribute to aggressive disease than the abundant neutrophils in favorable prognosis subgroups.

With regard to previously proposed roles of *TP63*, *IL1A*, and *CXCL1* in promoting neutrophil recruitment,^27^ our study supports an association between greater *CXCL1* expression and greater neutrophil abundance, consistent with a role for *CXCL1* in neutrophil recruitment. Expression of *TP63* and *IL1A* was associated with poor prognosis molecular subgroups, as previously reported,^4^, ^27^ but was not associated with neutrophil scores. Neutrophil infiltration into favorable prognosis subgroup tumors may, therefore, be driven by mechanisms independent of *TP63* and *IL1A*.

Overall, our study highlights neutrophil recruitment and activity as subjects of interest for further study. With recent attention to the role of stromal factors in tumor progression and stromal targeted therapies,^11^, ^34^ it may be of particular interest to explore how interventions modifying neutrophil infiltration may impact chemosensitivity. We note that the prognostic associations in our study were independent of differences in treatment.

In contrast to prior studies, the present study does not support an association between stromal maturity and overall survival.^12^, ^13^, ^14^ Although stromal maturity is a well validated prognostic factor for colorectal cancer,^24^ classifying tumors by their most immature stromal area, as was proposed for colorectal cancer metastases, would result in nearly all samples in our study being classified as having immature stroma. Samples with >10% extracellular mucin were previously found to be enriched in the classical type^2^; however, we saw no correlation between the presence or absence of mucin and tumor subgroup.

In summary, we identify for the first time the independent prognostic significance of combined gland formation and neutrophil infiltration in PDAC. We find these histologic features to correlate with molecular subgroups, providing a practical means of stratifying tumors into biologically meaningful subgroups associated with differences in survival and treatment response. These insights into the clinical and biological significance of histologic features may be used to stratify cases for both mechanistic studies and therapy development.

## CONFLICTS OF INTEREST

D.F.S. reports consultant fees from Robarts Clinical Trials Inc, unrelated to the work presented. D.J.R. disclosures include research funding and honoraria from Bayer, and honoraria from Servier, Celgene, Taiho, and Ipsen. J.L. declares honoraria for academic talks from Roche Canada, BI Canada, AstraZeneca Canada, and research grants from Roche Canada, Pfizer Canada, and AstraZeneca Canada. The remaining authors have no conflicts of interests to declare.

## FUNDING INFORMATION

The remaining authors have no conflicts of interests to declare. This study was supported by Pancreas Centre BC and The University of British Columbia Department of Pathology Residency Training Program. Resources generated in the POG program were funded by the BC Cancer Foundation and Genome BC (project B20POG). Resources generated through the PanGen program were funded by the BC Cancer Foundation, Pancreatic Cancer Canada, and the Terry Fox Research Institute (Project #1078).

## Supporting information

Supplementary Material

## Data Availability

Whole genome and transcriptome sequencing data are accessible through the European Genome‐468 phenome Archive (EGA; http://www.ebi.ac.uk/ega/) under study accession number #EGAS00001001159.
