# Supplementary material for: Tumor infiltrating neutrophils and gland formation predict overall survival and molecular subgroups in pancreatic ductal adenocarcinoma
Source: Cancer Med. 2020 Dec 28;10(3):1155–65. doi: 10.1002/cam4.3695 (PMC7897949; doi:10.1002/cam4.3695)
Supplement: Supplementary file 1 — Supplementary Material [file CAM4-10-1155-s001.pdf]

Supplemental Table 1: Demographics of patients with in-house frozen or FFPE sections

Demographic	n (%)
**Age**	
≤ 50	13 (26)
51-60	18 (36)
> 60	19 (38)
**Sex**	
Male	27 (54)
Female	23 (46)
**Extent of disease at diagnosis**	
Locally Advanced	4 (8)
Metastatic	46 (92)
**Grade**	
Well-differentiated	7 (14)
Moderately-differentiated	29 (58)
Poorly-differentiated	14 (28)
**Primary disease resected**	12 (24)
**First line chemotherapy**	
fluorouracil/oxaliplatin based	26 (52)
gemcitabine/paclitaxel based	22 (44)
capecitabine	1 (2)
none	1 (2)
**Immunotherapy treatment**	13 (26)
**Samples available for analysis**	
Only frozen	15 (30)
Only FFPE	3 (6)
Both frozen and FFPE	32 (64)

Supplemental Table 2: P-values from Fisher's exact test of the association between histologic features and the poor prognosis subtype of each gene expression-based classifier.

Histologic feature	p-value for association with poor prognosis subgroup on each classifier							
	Frozen section samples				FFPE validation samples			
	Moffitt	Collisson	Bailey	Karasinska	Moffitt	Collisson	Bailey	Karasinska
Stromal neutrophils (score ≤2 vs >2)	0.17	0.071	0.29	1.00	0.0014	0.0083	0.0083	0.13
Luminal neutrophils (score ≤2 vs >2)	0.034	0.071	0.071	0.30	0.0090	0.040	0.040	0.41
Gland formation (≤30% vs >30%)	0.014	0.0011	0.0011	0.33	0.070	0.021	0.0025	0.059
Gland formation ≤30% and stromal neutrophils ≤2	0.0019	0.00010	0.00010	0.11	0.0019	0.00081	$5.9x10^{-5}$	0.039
Gland formation ≤30% and luminal neutrophils ≤2	0.0019	0.00010	0.00010	0.11	0.00031	$7.5x10^{-5}$	$2.9x10^{-6}$	0.040
Gland formation ≤30% and luminal and stromal neutrophils ≤2	0.0022	0.00028	0.00028	0.050	0.00015	$8.9x10^{-5}$	$3.8x10^{-6}$	0.0076
Lymphocytes (low vs high)	0.070	0.14	0.14	0.70	0.54	0.74	0.21	1.00
Stromal maturity score (entirely immature vs partially mature)	1.00	0.13	0.30	0.70	0.76	0.52	0.52	0.70
Mucin (present vs absent)	0.074	0.16	0.16	1.00	1.00	0.75	0.75	0.71
Necrosis (present vs absent)	1.00	1.00	0.73	0.46	0.29	0.47	0.47	1.00

Supplemental Table 3: Univariate and multivariate Cox regression models of the association of histologic features with OS in the frozen section and FFPE in-house samples

Histologic Feature(s)	Frozen section samples				FFPE validation samples			
	Univariate		Multivariate		Univariate		Multivariate[1]	
	HR (95% CI)	P-value	HR (95% CI)	P-value	HR (95% CI)	P-value	HR (95% CI)	P-value
low stromal neutrophils	1.27 (0.57-2.83)	0.56	1.87 (0.75-4.65)	0.18	2.28 (1.06-4.92)	0.036	2.35 (0.96-5.75)	0.062
low luminal neutrophils	1.81 (0.82-4.02)	0.14	2.86 (1.10-7.49)	0.032	1.53 (0.72-3.30)	0.27	0.87 (0.30-2.51)	0.8
low stromal and luminal neutrophils	1.82 (0.87-3.82)	0.11	3.34 (1.34-8.30)	0.0095	1.60 (0.80-3.20)	0.19	1.61 (0.72-3.61)	0.25
≤30% gland formation	1.79 (0.88-3.63)	0.11	1.29 (0.43-3.92)	0.65	1.99 (0.99-4.01)	0.053	5.60 (1.84-17.06)	0.0025
≤30% gland formation and low stromal neutrophils	2.02 (1.02-4.00)	0.043	2.63 (0.84-8.23)	0.097	3.16 (1.56-6.42)	0.0015	3.51 (1.49-8.29)	0.0041
≤30% gland formation and low luminal neutrophils	2.61 (1.31-5.18)	0.0064	4.38 (1.31-14.64)	0.016	2.514 (1.26-5.03)	0.0090	3.07 (1.05-8.97)	0.040
≤30% gland formation and low combined neutrophils	2.55 (1.28-5.05)	0.0074	3.93 (1.33-11.63)	0.013	3.112 (1.53-6.33)	0.0017	3.58 (1.43-8.97)	0.0066

[1]Multivariate analysis included patient age and sex, tumor grade, extent of disease at diagnosis (i.e. metastatic vs locally advanced) whether the primary tumor was resected, and whether the patient received immunotherapy.
CI = confidence interval

Supplemental Table 4: Univariate and multivariate Cox regression models of OS in cases with versus without low gland formation and low combined neutrophils.

Samples	Univariate		Multivariate[1]	
	HR (95% CI)	P-value	HR (95% CI)	P-value
All combined frozen and FFPE in-house samples (n = 96)	2.89 (1.77-4.71)	2.1×10^{-5}	2.48 (1.37-4.51)	0.0029
Frozen and FFPE in-house sections of primary tumor (n = 17)	22.5 (2.34-215)	0.0070	48.68 (1.65-1433.90)	0.024
Frozen and FFPE in-house sections of metastatic tumor (n = 79)	2.64 (1.55-4.50)	0.00033	2.15 (1.14-4.07)	0.018
TCGA samples	3.41 (1.30-8.92)	0.0124	3.49 (1.33-9.18)	0.0112

[1]Multivariate analysis for in house samples included patient age and sex, tumor grade, extent of disease at diagnosis (metastatic versus locally advanced),whether the primary tumor was resected and whether the patient received immunotherapy. Multivariate analysis for TCGA sam ples included patient age and sex.

CI = confidence interval

Supplemental Table 5: The sensitivity and specificity of low gland formation and low combined neutrophils for the poor prognosis subgroup of the gene expression-based classifiers, calculated using in-house and TCGA samples.

Classifier	Poor prognosis subgroup	≤30% gland formation and low combined neutrophils			
		Frozen and FFPE in-house samples		TCGA samples	
		Sensitivity (%)	Specificity (%)	Sensitivity (%)	Specificity (%)
Moffitt	Basal-like	70	83	76	84
Collisson	Quasi-mesenchymal	79	82	54	60
Bailey	Squamous	82	84	64	68
Karasinska	Glycolytic	68	74	63	65

Supplemental Table 6: The sensitivity and specificity of high gland formation and high combined neutrophils for favorable prognosis subgroups of the gene expression-based classifiers, calculated using in-house and TCGA samples.

Classifier	Favorable prognosis subgroups	>30% gland formation high luminal and stromal neutrophils			
		Frozen and FFPE in-house samples		TCGA samples	
		Sensitivity (%)	Specificity (%)	Sensitivity (%)	Specificity (%)
Moffitt	Classical	27	94	24	100
Collisson	Classical and exocrine-like	26	96	21	96
Bailey	ADEX, immunogenic and progenitor	25	93	21	100
Karasinska	Cholesterogenic, quiescent and mixed	23	91	19	100

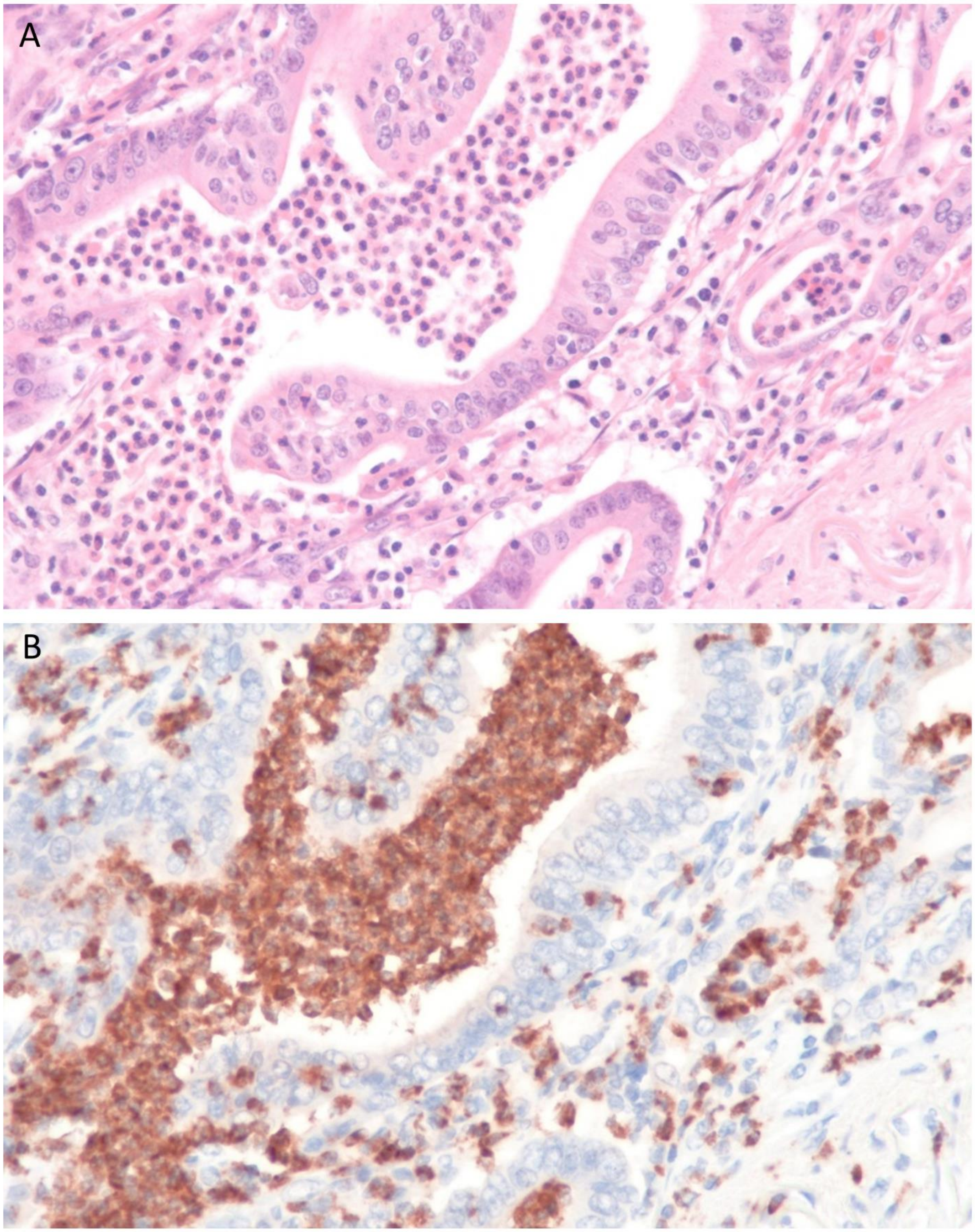

Supplemental Figure 1: **(A)** an H&E stained section of PDAC is confirmed to have high levels of neutrophil infiltration through **(B)** myeloperoxidase immunohistochemistry of an immediately adjacent section. Neutrophils are highlighted by myeloperoxidase immunostaining (brown).

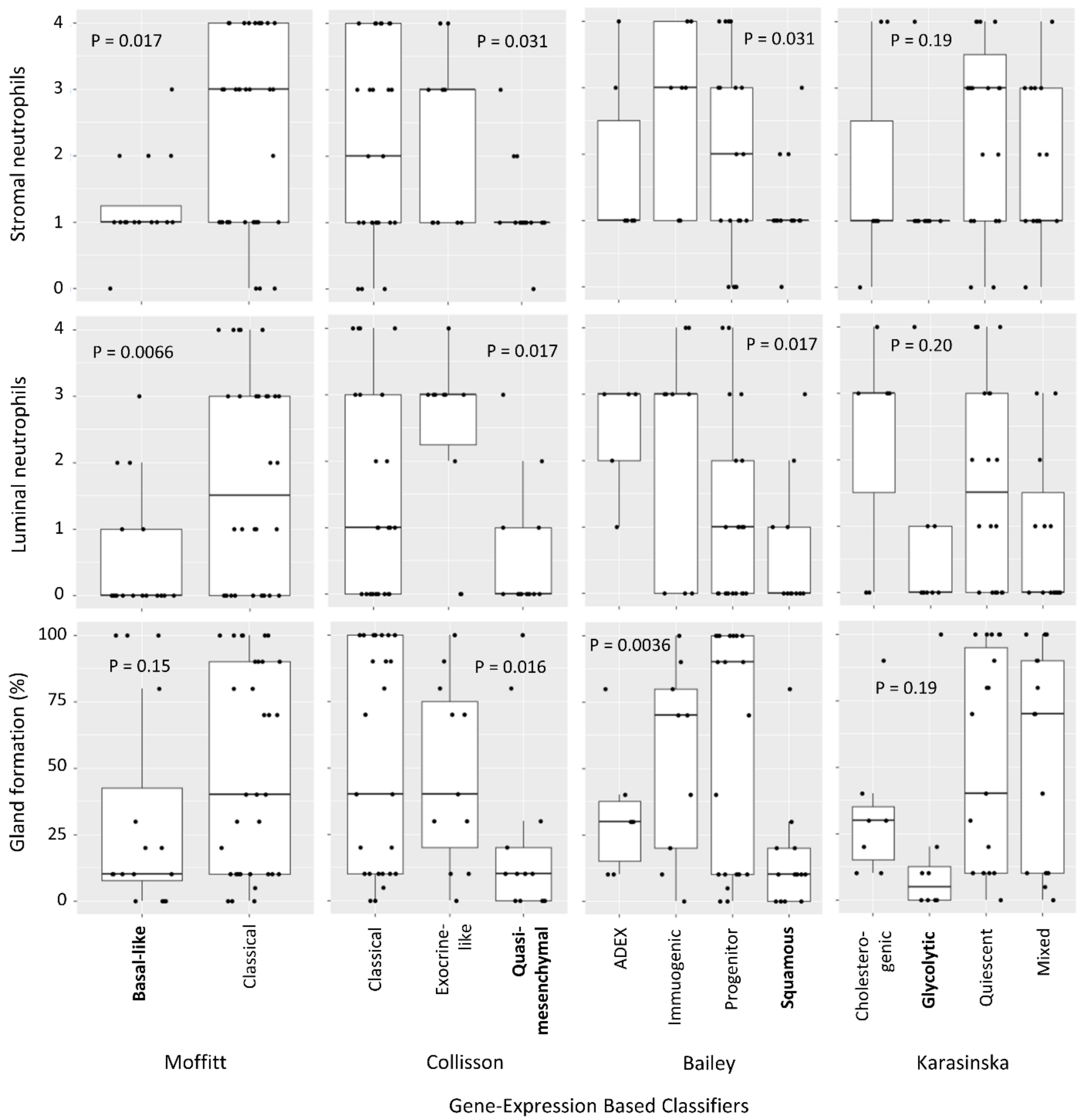

Supplemental Figure 2: When using the FFPE in-house validation samples (n = 49), neutrophil infiltration and gland formation tended to be associated with the basal-like, quasi-mesenchymal and squamous subtypes. P-values were calculated using the Wilcoxon-Mann-Whitney test comparing the poor prognosis subgroup (shown in bold) to the other subgroups. Boxes extend from the first to third quartile, with a line at the median. Points indicate scores for individual samples.

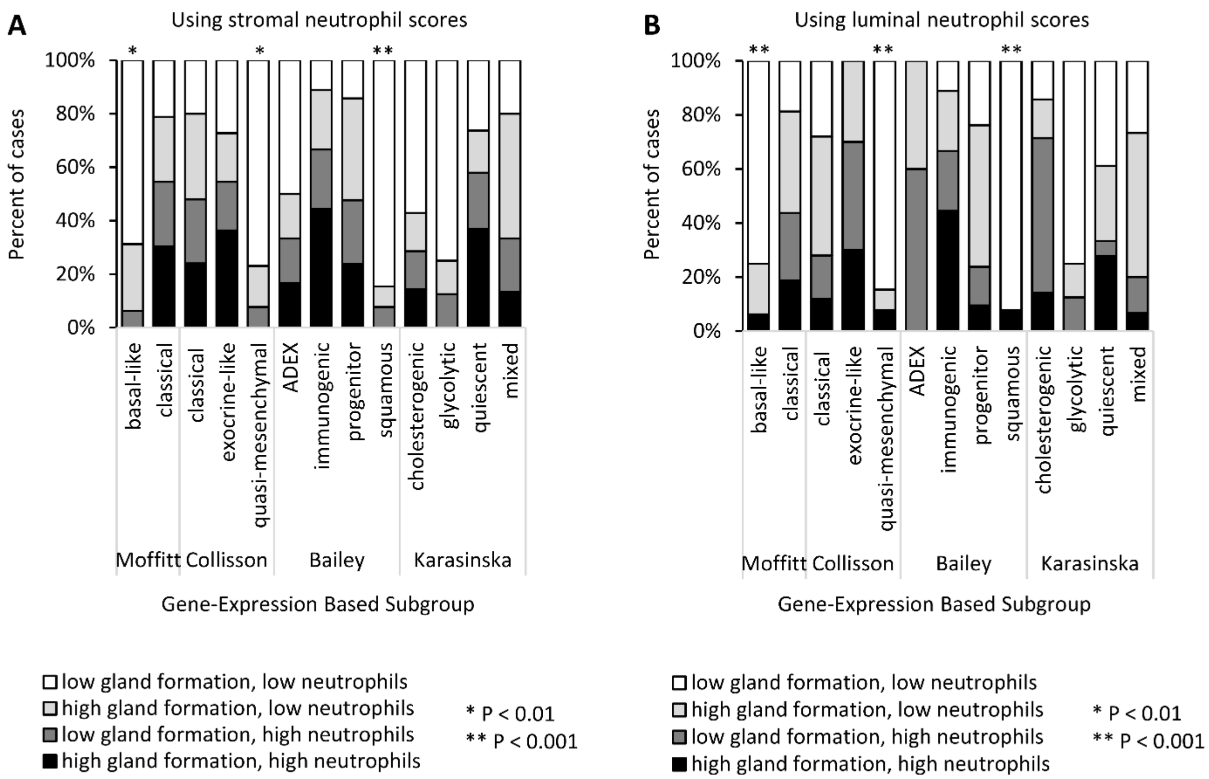

Supplemental Figure 3: The proportion of samples with low gland formation and low neutrophils (either **[A]** stromal or **[B]** luminal) in each subgroup of the gene expression-based classifiers, scored using the FFPE in-house validation samples (n = 49). Cases with both low gland formation and low neutrophils were significantly enriched in the basal-like, quasi-mesenchymal and squamous subtypes. Fisher's exact test P-values are shown. Low gland formation was defined as ≤30% and low neutrophils as a score of ≤ 2 out of 4.

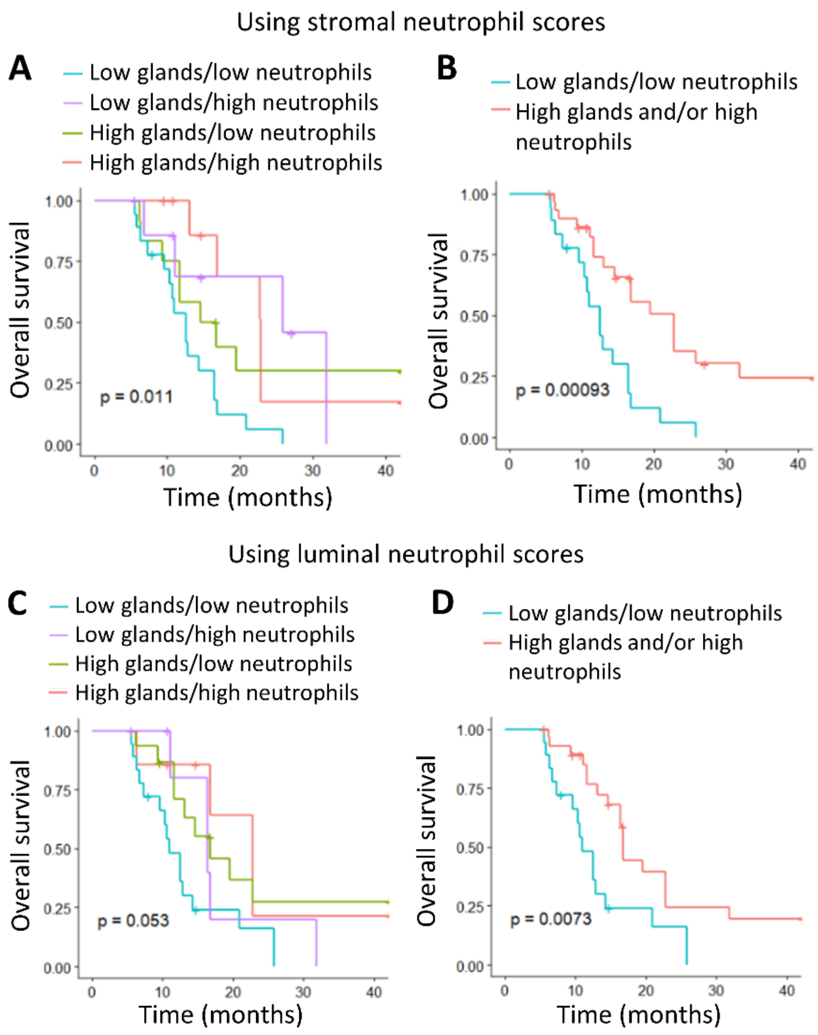

Supplemental Figure 4: Kaplan-Meier curves for OS stratified by tumor gland formation and neutrophil infiltration, scored in FFPE in-house validation samples (n = 49). Low gland formation was defined as ≤ 30% and low neutrophils as ≤ 2 out of 4. P-values were calculated using log-rank tests.

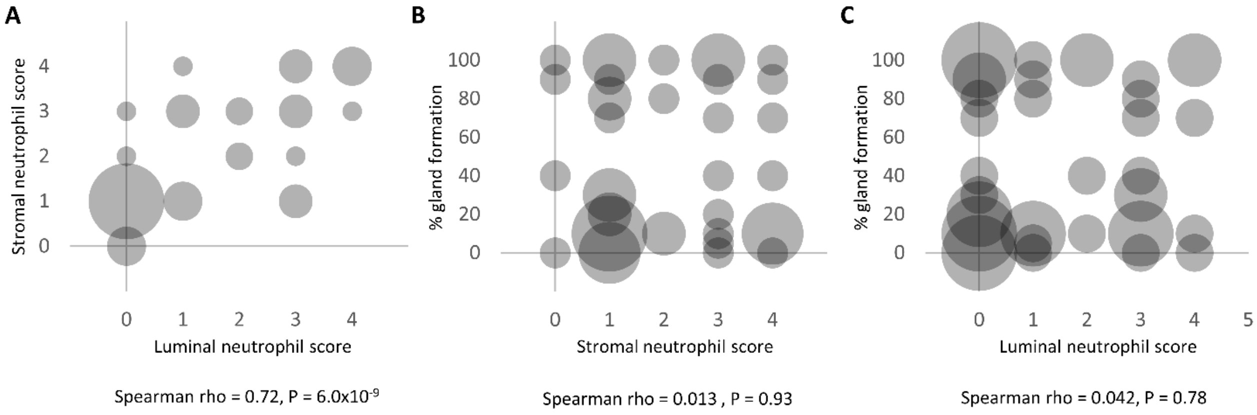

Supplemental Figure 5: Correlation between percent gland formation, stromal neutrophil score and luminal neutrophil score in the same sample, using FFPE in-house validation samples. **(A)** Stromal and luminal neutrophil scores were well correlated, whereas **(B,C)** gland formation was poorly correlated with neutrophil scores. Bubble size is proportional to the number of case with a given score combination, ranging up to 15 for **(A)**, up to 6 for **(B)**, up to 4 for **(C)**. Spearman correlation (rho) and P-values are shown.

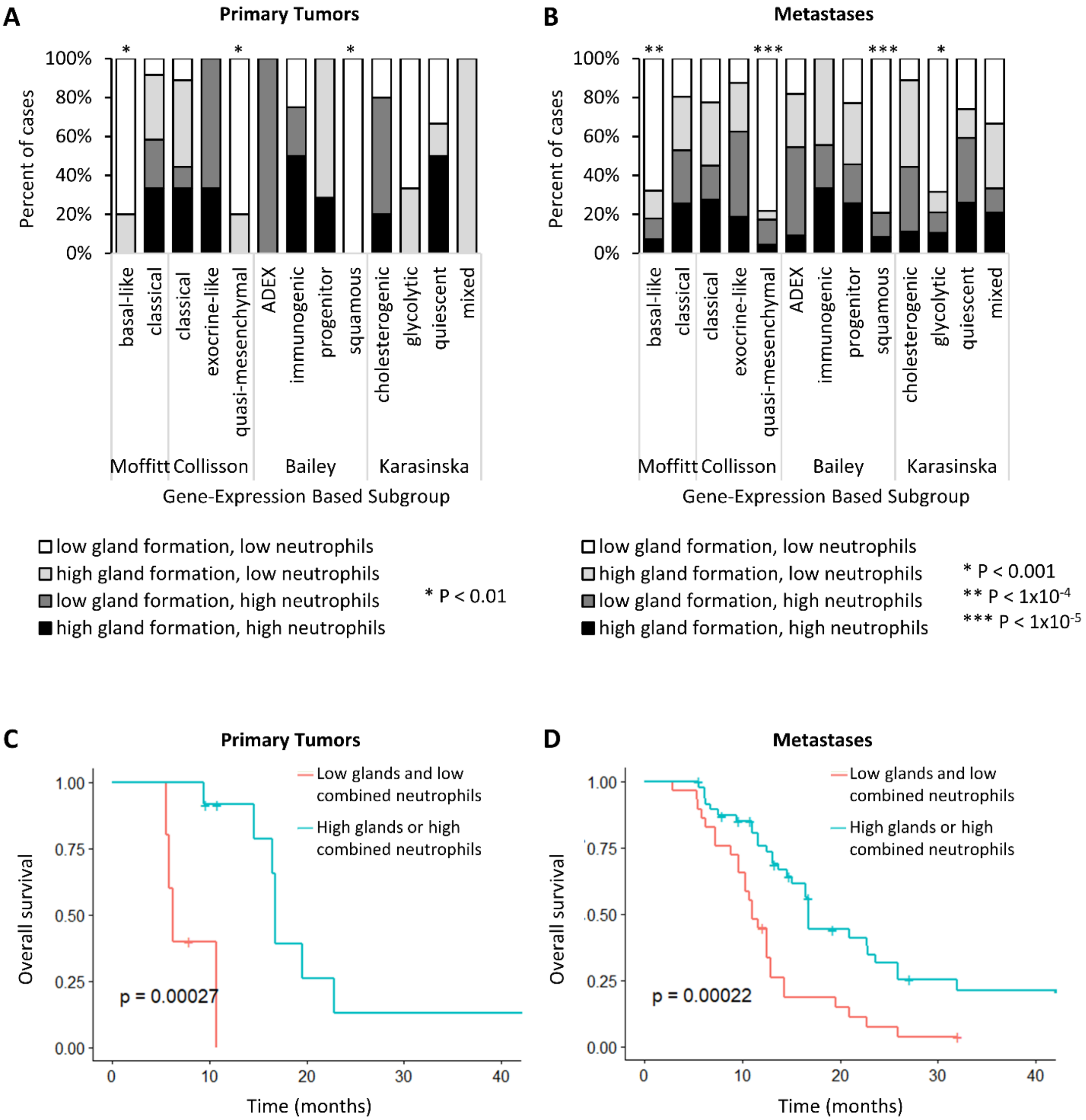

Supplemental Figure 6: Subgroup and survival associations of low gland formation and low combined neutrophils in pooled frozen and FFPE in-house samples, divided by **(A,C)** primary (n = 17) and **(B,D)** metastatic sites (n = 79). **(A, B)** The proportion of samples with low gland formation and low combined neutrophils in each subgroup of the gene expression-based classifiers. Fisher's exact test P-values are shown. **(C, D)** Kaplan-Meier curves for OS stratified by tumor gland formation and neutrophil infiltration. P-values were calculated using log-rank tests. Low gland formation was defined as ≤ 30%. Cases scoring ≤ 2 out of 4 for both stromal and luminal neutrophils were considered to have 'low combined neutrophils'.

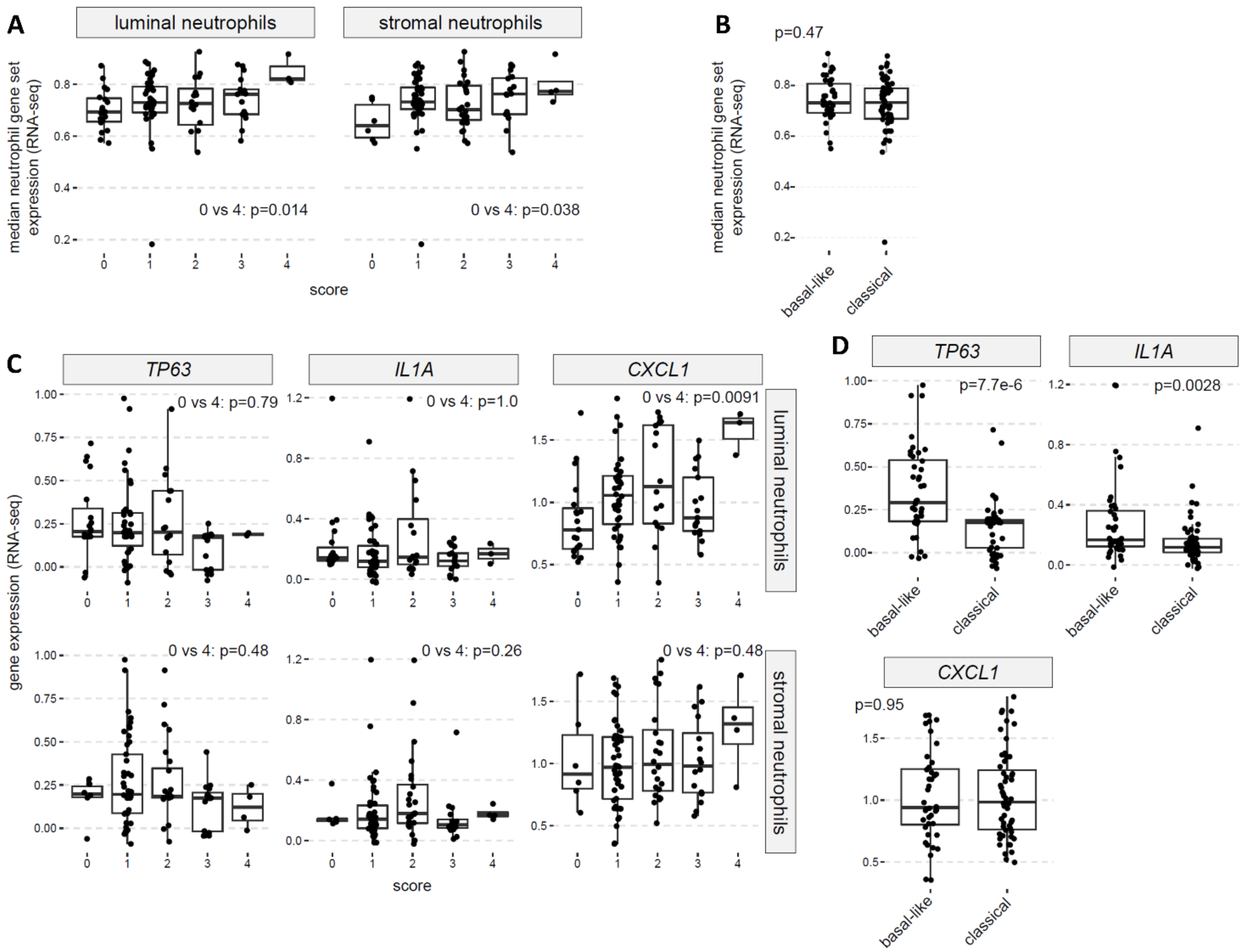

Supplemental Figure 7: Association of inflammatory gene expression signatures with histologically identified neutrophils and Moffitt molecular subtypes. **(A)** Median expression of neutrophil-associated genes (GO_NEUTROPHIL_MEDIATED_IMMUNITY; n=22) across samples of varying luminal (left) and stromal (right) neutrophil histopathology scores. Neutrophil-associated gene expression is significantly ($p<0.05$) higher in samples with a score of 4, for both luminal and stromal scores. **(B)** Median expression of neutrophil-associated genes compared between basal-like and classical samples. **(C)** *TP63*, *IL1A* and *CXCL1* gene expression across samples of varying luminal (top) and stromal (bottom) neutrophil histopathology scores. **(D)** *TP63*, *IL1A* and *CXCL1* gene expression compared between basal-like and classical samples. *TP63* and *IL1A* show significantly ($p<0.05$) higher expression in basal-like samples. Wilcoxon mean-rank sum p values are shown.
